# The Role of Cardiac Magnetic Resonance in Aortic Stenosis and Regurgitation

**DOI:** 10.3390/jcdd9040108

**Published:** 2022-04-04

**Authors:** Marco Guglielmo, Chiara Rovera, Mark G. Rabbat, Gianluca Pontone

**Affiliations:** 1 Centro Cardiologico Monzino IRCCS, 20138 Milan, Italy; m.guglielmo@umcutrecht.nl (M.G.); roverachiara@gmail.com (C.R.); 2 Division of Cardiology, Loyola University of Chicago, Chicago, IL 60611, USA; mrabbat@lumc.edu; 3 Edward Hines Jr. VA Hospital, Hines, IL 60141, USA

**Keywords:** cardiovascular magnetic resonance, aortic valve, aortic stenosis, aortic regurgitation, transcatheter aortic valve implantation

## Abstract

Cardiac magnetic resonance (CMR) imaging is a well-set diagnostic technique for assessment of valvular heart diseases and is gaining ground in current clinical practice. It provides high-quality images without the administration of ionizing radiation and occasionally without the need of contrast agents. It offers the unique possibility of a comprehensive stand-alone assessment of the heart including biventricular function, left ventricle remodeling, myocardial fibrosis, and associated valvulopathies. CMR is the recognized reference for the quantification of ventricular volumes, mass, and function. A particular strength is the ability to quantify flow, especially with new techniques which allow accurate measurement of stenosis and regurgitation. Furthermore, tissue mapping enables the visualization and quantification of structural changes in the myocardium. In this way, CMR has the potential to yield important prognostic information predicting those patients who will progress to surgery and impact outcomes. In this review, the fundamentals of CMR in assessment of aortic valve diseases (AVD) are described, together with its strengths and weaknesses. This state-of-the-art review provides an updated overview of CMR potentials in all AVD issues, including valve anatomy, flow quantification, ventricular volumes and function, and tissue characterization.

## 1. Introduction

Aortic valve disease (AVD) affects approximately 0.9% of the general population [1], with a progressive increase in prevalence with advancing age [2].

Although echocardiography still represents the first-line technique to assess the aortic valve, cardiovascular magnetic resonance (CMR) imaging is emerging as a method able to provide a comprehensive evaluation of many aspects of aortic valvulopathy.

CMR is a non-invasive, multiplanar, and high-spatial-resolution imaging technique. It offers a robust alternative for assessing the severity of aortic stenosis (AS) [3], is superior to echocardiography in the grading of aortic regurgitation (AR) [4], and can characterize the anatomy of the entire thoracic aorta. CMR represents the current gold standard for evaluating ventricular volumes, mass, and function, and can identify left ventricular (LV) remodeling due to aortic valvulopathies. Furthermore, it has the advantage of characterizing the myocardial tissue, which can provide important prognostic information [5].

The aim of this review is to provide an updated overview of CMR in AVD. In this paper, we will first review the CMR sequences commonly used to assess patients with aortic valvulopathy. Then, we will discuss the role of CMR in AS and AR. Next, we will focus on the application of CMR in patients requiring transcatheter aortic valve prosthesis implantation (TAVI).

## 2. Sequences Used to Assess Patients with Aortic Valvulopathy

### 2.1. Assessment of Aortic Valve Anatomy

The steady-state free precession (SSFP) pulse sequence is commonly used for the assessment of valve morphology and function [6,7]. Image acquisition is gated to the ECG, and each slice is attained during a single breath-hold of 5–8 s. SSFP sequences allow high contrast between the bright blood pool and adjacent regions, with a high signal-to-noise ratio. SSFP generates two-dimensional (2D) cine images for the evaluation of the morphology and motion of the aortic valve. Furthermore, planimetry enables direct sizing of valve orifice area by arranging the slice image at the valve tips. A sequence of short-axis or long-axis images are obtained with a spatial resolution of 1.2–1.5 mm and temporal resolution of 20–40 ms. Nevertheless, partial volume effects and failure to identify thin structures/small vegetations are contingent limitations due to rather thick slices of 5 to 8 mm. Consequently, careful orientation of the imaging slice perpendicular to the valve plane and the use of a slice thickness of 4 to 6 mm are necessary to reduce these limits. Moreover, cine SSFP imaging is affected by arrhythmias.

### 2.2. Flow

Cine sequences enable visualization of post-stenotic and regurgitant blood flow. Qualitative analysis relies on the evaluation of signal void artifact, which results from intravoxel spin dephasing due to turbulent flow. Gradient echo imaging (GRE) [8], a former “bright blood” cine imaging sequence, has the advantage of a more intense spin dephasing effect and, therefore, an improved sensitivity in flow anomaly detection. Flow voids have to be analyzed in multiple planes to avoid incomplete characterization and inaccurate semi-quantitative assessments.

Quantitative analysis of flow velocity can be performed using through-plane phase contrast (PC) velocity mapping [9]. PC pulse sequences center on the principle that applications of velocity-encoding (VENC) gradient pulses induce phase shifts in moving protons that are directly proportional to their velocity along the direction of the magnetic field gradient. The net phase of moving protons is proportional to the velocity of blood and can be displayed as a phase map. Flow volume is obtained by multiplying the velocity within each pixel by the area and a flow-time graph is developed over one cardiac cycle. The imaging slice is usually placed just above the aortic valve. One forewarning for quantifying flow is that the position of the slice is fixed in space, whereas the valve moves. On account of this, the velocities are not sampled at the same anatomic location throughout the cardiac cycle. However, this is usually not a matter of importance for the quantification of the aortic valve flow [10]. PC flow assessment can be achieved with free-breathing or breath-hold techniques. PC data are collected over several heartbeats; for this reason, the accuracy of flow measurements is diminished if an irregular rhythm is present. Several vendors employ arrhythmia rejection algorithms, with the exclusion of beats with very divergent R-R intervals. Rejecting a lot of beats, however, substantially increases scan time, making breath-holding troublesome. Real-time single-beat acquisition may represent the answer to this problem [11,12]. Furthermore, non-breath-hold flow sequences with navigator-based motion suppression are advised for their minor background flow offset errors [13]. Temporal resolution of CMR flow measurement is 25–45 ms; therefore, for high flow velocities of brief duration, CMR may underrate peak velocity. However, even so, most flow measurements are feasible [14]. Another limitation of flow analysis is the existence of positive or negative phase offset errors, due to local turbulent currents [15]. This reduces the accuracy for velocities greater than 3.5 m/s. Nevertheless, the important advantages of this method are easy measurement, no geometric assumptions, no contrast agent application, and short investigation time. Post-acquisition correction methods, such as scanning a stationary gel phantom, may improve the reliability of flow quantification [16]. Moreover, advances in machine learning have notably enhanced automated processing [17].

### 2.3. Imaging of the Thoracic Aorta

CMR gives the possibility to characterize the anatomy of the thoracic aorta; aortic root dilatation is associated with functional AR, whereas post-stenotic remodeling occurs in AS, especially in bicuspid aortic valves. In patients scheduled for aortic valve intervention, it is fundamental to provide aortic measurements. One of the most used tools is contrast-enhanced MR angiography (CEMRA), which is usually non-ECG-triggered [18]. Thus, it produces images with a certain amount of blurring which is more pronounced at the aortic root level.

A three-dimensional (3D) self-navigated free-breathing high-resolution whole-heart CMR sequence with either end-systolic or diastolic gating [19] grants a high isovolumetric spatial resolution together with achieving the self-navigating readout at each heart beat and keeping uniformity in the sampling of the whole chest scan. This technique allows a contrast-free, ductile retrospective multiplanar reconstruction (MPR) of the image plane perpendicular to the vessel’s axis for very sharp rendering of the aortic root and determination of diameters.

Four-dimensional (4D) flow or time-resolved 3D phase-contrast CMR is applied for the synchronous evaluation of morphometry and flow parameters along the thoracic aorta. It holds the possibility to measure non-laminar flow in any direction in space during the cardiac cycle [20]. Velocity measurements are obtained in an entire volume of interest, permitting blood flow quantification during post-processing in any desired plane. Thus, 4D flow MRI is appropriate to visualize and quantify eccentric and dynamic flows [21]. It can also be used to determine regional aortic wall shear stress from near-wall blood flow velocity gradients [22]. There are some limitations concerning 4D flow [23]. It can only represent the sum or average of hemodynamic events that repeat every cardiac cycle. Therefore, it is difficult to capture other transient flows and fluctuations related to respiration. Another technical limitation is that only one VENC can be set for one data acquisition.

### 2.4. Ventricular Volume and Function

CMR is the gold-standard imaging technique to evaluate LV volume, mass, and function [24]. Accurate assessment is essential to decide the timing for intervention. SSFP techniques have been well validated for this purpose [25]. Ventricular volumes are calculated from a short-axis stack of 6–8 mm thick slices with an interslice gap of 4 mm. CMR-derived myocardial feature tracking (CMR-FT), based on optical flow methods, is able to detect specific patterns of features or image irregularities and track them along the cardiac cycle, especially in the endocardial border. Myocardial deformation can be evaluated using CMR-FT applied to routine cine-CMR images also in patients with AVD [26].

### 2.5. Tissue Characterization

Late gadolinium contrast-enhanced (LGE) imaging is deemed to be the reference standard to quantify myocardial replacement fibrosis and scar. Standard LGE sequences obtained 10 to 15 min after contrast agent injection. Furthermore, inversion recovery gradient echo sequences are used, consisting of an inversion pulse to suppress normal myocardium and a T1-weighted GRE acquisition. In regions with higher gadolinium concentration, T1 time is shorter than in adjacent areas and shows high signal intensity on LGE images. Normal myocardial tissue will appear darker compared with the bright signal of the damaged myocardium where gadolinium washout is delayed [27]. Another technique that allows a more quantitative approach for tissue characterization is T1 mapping, used to calculate the T1 relaxation times of myocardial tissue, displaying them on a parametric map so that each pixel has a T1 value. An increase in native T1 (without the use of contrast agents) may be caused by the presence of edema, fibrosis, or protein accumulation. Post-contrast T1 mapping, in combination with hematocrit levels, is essential for extracellular volume (ECV) quantification. ECV mapping can detect and monitor collagen build-up in the myocardium, providing a quantitative tool to evaluate diffuse myocardial fibrosis or an extracellular compartment increase [28]. Several protocols have been proposed for the acquisition of T1 maps, such as the modified Look–Locker inversion recovery (MOLLI) technique [29,30].

## 3. CMR Assessment of Aortic Valve Stenosis

AS is the most common valvulopathy in developed countries with a prevalence constantly increasing due to rising life expectancy [31,32]. Transthoracic echocardiography (TTE) remains the first-line test in patients with AS, providing anatomy depiction of the aortic valve and hemodynamic parameters to define the degree of stenosis. Moreover, TTE is valuable for the assessment of aortic dimensions, LV remodeling, and associated valve diseases, as well as to rule out subvalvular or supravalvular stenosis. Transesophageal echocardiography (TEE) is useful in the presence of suboptimal acoustic windows, particularly to define valve anatomy [33]. In the presence of discordant echocardiographic parameters, computed tomography (CT), by measuring the calcium load [34] and determining the dimensions of the LV outflow tract [35], can be useful to confirm AS severity. Furthermore, CT may provide detailed anatomical information about the aortic annulus and the aorta, as well as regarding the feasibility of peripheral access in patient candidates for TAVI [36].

Even though less utilized in clinical practice, CMR offers several advantages in patients affected by AS, allowing a non-invasive, multiplanar, radiation-free, and high-resolution assessment of valvular anatomy and severity of stenosis, coupled with a thorough functional evaluation. Furthermore, compared to other modalities, CMR offers the unique asset of myocardial tissue characterization. In patient candidates for TAVI, as described later, CMR represents an alternative tool to CT for procedural planning in subjects with contraindications to contrast agents.

### 3.1. Valvular Anatomy and Degree of Stenosis

**Figure 1:**
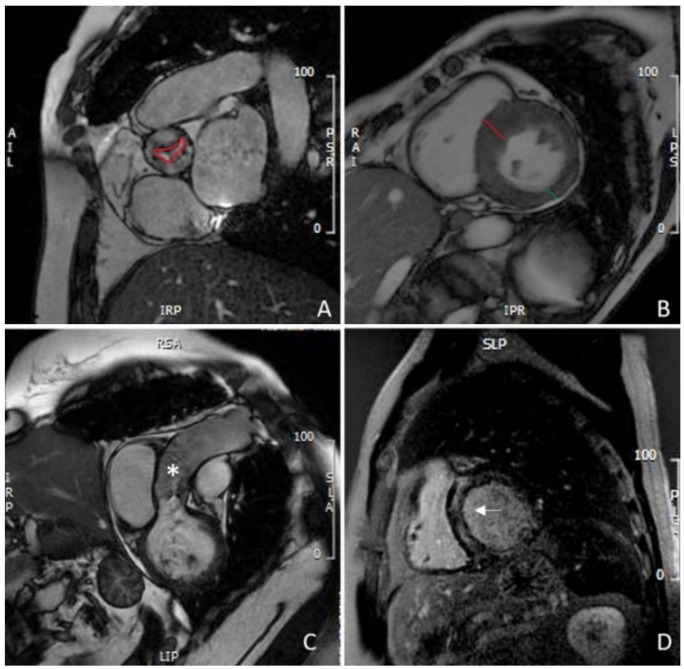
CMR evaluation of aortic stenosis: (**A**) planimetry of aortic valve (red line); (**B**) measurement of IVS (red line) and PW thickening (green line), showing asymmetric hypertrophy of the IVS; (**C**) assessment of thoracic aorta (asterisk); (**D**) mid-wall LGE of the IVS. CMR: cardiovascular magnetic resonance; IVS: interventricular septum; PW: left ventricle posterolateral wall; LGE: late gadolinium enhancement.

In patients with non-diagnostic TTE due to poor acoustic windows, CMR can be used for aortic valve anatomy assessment and to determine the degree of stenosis. Assessment of AS severity by CMR utilizes two parameters: planimetry of the valve area (Figure 1A) and peak velocity/gradient across the aortic valve [37].

The latter, different from Doppler echocardiography, is time-consuming and tends to underestimate the transvalvular gradients secondary to intravoxel dephasing errors in presence of high-velocity flows [38]. On the contrary, CMR planimetry of the aortic valve area (AVA) offers a noninvasive and reproducible technique to evaluate AS, with a high correlation with measurements obtained with TEE, which are particularly relevant in the presence of inadequate acoustic windows [3]. Although not routinely used in clinical CMR, 4D flow offers an alternative method for non-invasive assessment of AS. Four-dimensional flow has the advantage of identifying the true peak velocity across the 3D aortic valve and also overcomes many of the problems of echocardiographic measurement, such as Doppler misalignment, flow, and geometric assumptions. The identification of the maximum velocity in a 3D space is a major advantage, not only for Doppler TTE but also the current standard PC CMR methods for AS assessment, which are recognized to underestimate velocities [39]. Four-dimensional flow also gives the opportunity to derive advanced hemodynamic measures, such as vorticity and helicity, wall shear stress, flow displacement, pressure gradients, viscous energy loss, and turbulent kinetic energy. These new metrics are used in research applications, but there is growing evidence that flow changes may play an active role in the development of AS-mediated aortopathy (Figure 1C), such as dilatation, aneurysm, or dissection. Four-dimensional flow may thus have the potential to inform individualized treatment decisions for an optimized patient outcome [40].

### 3.2. LV Remodeling

LV remodeling in the setting of AS starts as a compensatory process to maintain wall stress, but often it progresses to a maladaptive response characterized by myocyte hypertrophy, interstitial fibrosis, and apoptosis. Although LV reverse remodeling occurs after aortic valve replacement (AVR), the intervention is often performed late after irreversible maladaptive LV remodeling and fibrosis [41]. Dweck and al. demonstrated that in patients with moderate and severe AS, LV adaption patterns and the degree of hypertrophy do not closely correlate with the severity of valve narrowing and that asymmetric patterns of wall thickening are common with a considerable overlap in the appearance with hypertrophic cardiomyopathy [42]. More recently, the same group showed that asymmetric wall thickening is associated with increased myocardial injury, left ventricular decompensation, and adverse events. Importantly, asymmetric wall thickening was identified more frequently with CMR than with echocardiography [43]. Whether early replacement of the aortic valve may be beneficial for patients with asymmetric wall thickening is unknown and requires further investigations. More recently, Hwang et al. demonstrated that longitudinal global strain (GLS) measured by CMR-FT is predictive of LV mass index regression after AVR in patients with AS [44]. An example of LV remodeling with asymmetric hypertrophy of the interventricular septum is shown in Figure 1B.

### 3.3. Tissue Characterization

Myocardial fibrosis is a hallmark of severe AS and has an important prognostic role. Three main patterns have been described: endocardial fibrosis, microscars (mainly in the subendomyocardium), and diffuse interstitial fibrosis [45].

Although the gold standard for assessing myocardial fibrosis is histology on endomyocardial biopsy, CMR is able to non-invasively assess the presence of both focal and diffuse fibrosis. Focal non-ischemic fibrosis, often identified as an area of mid-wall LGE (Figure 1D), is frequent, correlates with disease severity, and is an independent predictor of mortality [46,47,48,49,50].

Similarly, diffuse fibrosis, identified with native T1 and ECV, is a relevant risk marker in patients with AS. Lee et al. showed that high native T1 value on non-contrast T1 mapping CMR is an independent predictor of adverse outcome in patients with significant AS [51]. More recently, Everett et al. demonstrated that in patients with severe AS undergoing AVR, diffuse myocardial fibrosis quantified with ECV by CMR T1 mapping is an independent predictor of all-cause mortality [52].

## 4. CMR Assessment of Aortic Valve Regurgitation

Recent guidelines suggest that CMR is indicated for AR evaluation when echocardiographic images are suboptimal, echo parameters are discordant, disagreement subsists between clinical assessment and echocardiographic grading, inadequate echocardiographic measurements of LV volumes and systolic function are obtained in patients with moderate/severe AR, and insufficient aorta evaluation by echocardiography is achieved in patients with bicuspid aortic valve [53].

CMR assessment of AR is advisable due to the high degree of accuracy for measurement of LV volumes and function as well as aortic regurgitant volumes [6,54]. This is particularly useful for serial measurements with high reproducibility, providing information about disease progression [4].

### 4.1. Valvular Anatomy and Degree of Regurgitation

**Figure 2:**
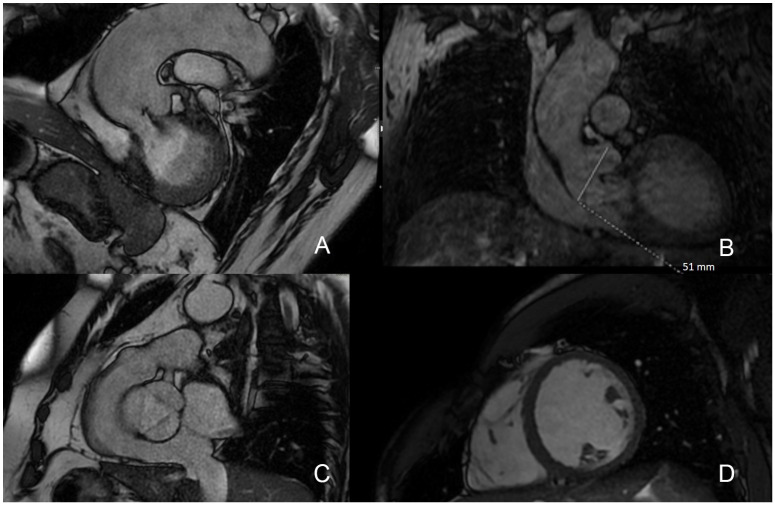
CMR SSFP sequences for AR assessment: (**A**) identification of regurgitant flow as signal void artifact; (**B**) assessment of aortic root; (**C**) aortic valve morphology evaluation; (**D**) left ventricle short-axis view. CMR: cardiovascular magnetic resonance; SSFP: steady-state free precession; AR: aortic regurgitation.

CMR assessment of AR initiates from the visual inspection of the aortic valve, aortic root, LV, and LV outflow tract structure and function with SSFP (Figure 2A). Valve morphology (e.g., bicuspid/tricuspid) and pathology (e.g., leaflet prolapse, endocarditis) are of particular interest to help determine mechanisms of AR. To study the morphology of the aortic valve, a single cine image placed at the tips of the cusps is usually sufficient (Figure 2C), but to measure the AVA, a stack of cines covering the aortic valve is generally required. Nonetheless, small vegetations in infective endocarditis and valvular masses are not always accurate by CMR due to constraints of spatial resolution and the non-real-time image acquisition over several cardiac cycles, which may miss structures with asynchronous mobility [55]. An evaluation of the aortic root (Figure 2B) can help to identify the cause of AR (e.g., hypertension, aortic dissection, and Marfan syndrome), as well the requirements for aortic root repair/replacement alongside AVR. As described above, different techniques can produce precise images and the possibility to measure diameters of the thoracic aorta [56].

A preliminary evaluation of the severity of AR can be achieved by visualization of the signal void of the regurgitant jet on cine imaging (Figure 2A). A narrow jet width suggests mild regurgitation, while a wide jet suggests more severe regurgitation. However, this method is subject to many potential inaccuracies; the size of the jet may not necessarily correlate to the severity of regurgitation, since it is caused by the local acceleration of the flow and does not directly reflect the regurgitant volume. This technique is not recommended for accurate evaluation [57]. Likewise, the regurgitant orifice area measured directly by planimetry and the calculation of the regurgitant jet area or length are not reliable indices of disease severity and are therefore not usually performed. GRE cine sequences are a useful addition when higher temporal resolution and higher sensitivity to flow alterations are desired [8].

**Figure 3:**
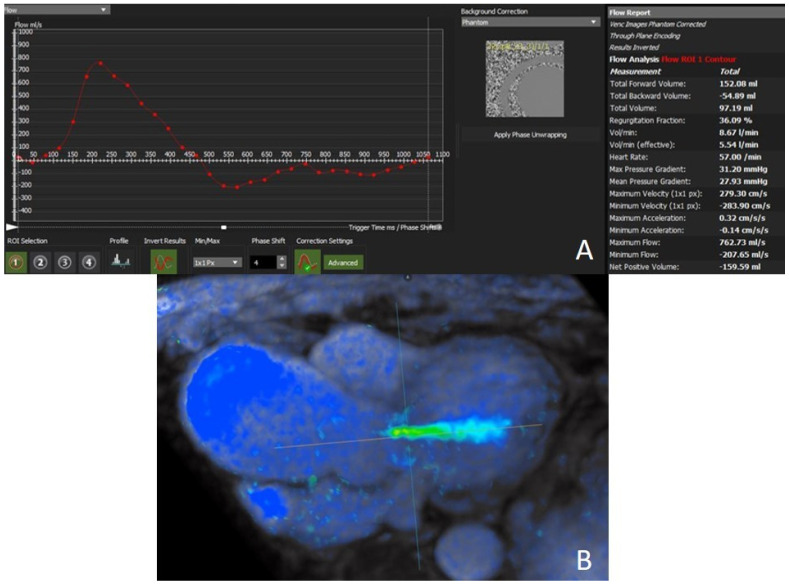
Aortic regurgitant flow quantification: (**A**) phase contrast imaging; (**B**) 4D flow technique.

However, the most commonly used method to quantify AR is through-plane PC imaging (Figure 3A), which calculates forward and reverse flow per cardiac cycle and compares aortic versus pulmonary forward stroke volumes [58]. Combining the flow curves, stroke volume (total forward flow), cardiac output (stroke volume x heart rate), regurgitant volume (total backward flow), and the regurgitant fraction (regurgitant volume/stroke volume) are inferred.

Regurgitant fraction and regurgitant volume are independent predictors of outcome in patients with AR. A regurgitant fraction of >33% and a regurgitant volume threshold of >42 mL have been shown to predict the likelihood of requiring surgery (mean follow-up of 2.6 years). No patients with a regurgitation fraction <26% progressed to surgery [59]. Moreover, Harris et al. found that a regurgitant fraction of >37% and a regurgitant volume of >50 mL had a sensitivity of 100% and specificity of 75% for requiring valve surgery during the 4-year follow-up [60]. Another study also showed a CMR-derived regurgitant fraction of >30% to best correlate with grade 4+ AR using echocardiography [61]. Gelfand et al. found that CMR regurgitant fraction thresholds for AR that had maximal agreement with echo were mild ≤15%, moderate 16%–27%, and severe >27% [62]. Other employed regurgitant fraction cut-offs are mild-AR (<20%), moderate AR (20–40%), and severe AR (≥40%) [58]. More research is needed to define the optimal cut-offs for surgery using CMR. However, these numbers are noticeably lower than the cut-off for severe AR used in echocardiography.

The presence of holodiastolic retrograde flow (HRF) in the descending aorta can also be assessed easily by PC imaging. HRF on CMR was strongly and independently associated with heart failure, hospitalization, and cardiovascular death [4].

Four-dimensional flow MRI is an emerging tool for the assessment of AR (Figure 3B). The advantages of 4D flow can be summarized as follows: 3D anatomical, functional, and flow data; free-breathing technique; retrospective analysis of any flow type (e.g., laminar or non-laminar) in any direction, balancing the longer duration of the sequence; visualization of complex or eccentric flows; retrospective tracking of one or more jets to avoid underestimating the regurgitant fraction; assessment of internal validity (e.g., by comparing values in the pulmonary artery with those calculated in the aorta); and identification of HRF in the descending aorta [63].

### 4.2. LV Remodeling and Myocardial Fibrosis

Accurate LV volumes with CMR are fundamental to clinically assess the impact of AR (Figure 2D). In a multi-center observational study LV, an end-diastolic volume (EDV) of >246 mL predicted the development of a class I guideline indication for surgery [59]. Furthermore, a composite of LV EDV and regurgitant fraction was advised as a powerful discriminator for the likelihood to progression to surgery [59].

Pressure and volume overload exerted on the LV by AR induce interstitial fibrosis, characterized by increased fibronectin and glucosamine deposit with altered collagen organization [64]. Replacement fibrosis has also been described with CMR. In a study that included 26 patients with severe AR, LGE was present in 69% of subjects, mostly following a multifocal pattern, and the correlation between LGE and histology was strong (*R* = 0.70, *p* < 0.001) [65]. Malahfji et al. demonstrated that myocardial scar was present in a third of 392 patients with AR, and was associated with mortality in multivariable analysis. In patients with scars, AVR was associated with better outcome as compared to medical treatment [47]. Sparrow et al. examined myocardial T1 values before and after gadolinium contrast administration in 8 patients with severe AR and 15 normal controls. AR patients had significantly increased post-contrast T1 values in segments with impaired contractility compared to the controls (510 ms vs. 476 ms, *p* = 0.001), implying the presence of expanded interstitial fibrosis [66]. In another study that included nine patients with severe AR who underwent AVR, ECV quantified on three Tesla CMR was robustly correlated with the amount of interstitial fibrosis on histology (*R* = 0.79, *p* = 0.011) [67]. CMR-FT myocardial deformation measurements were found impaired in patients with AR who failed to meet surgical indication. GLS decreases early in the progression of the disease and is a marker of AR severity, while radial (GRS) and circumferential strain (GCS) worsen later but predict a poor prognosis, mainly the need of AVR [68]. Moreover, in 14 patients with chronic severe AR, myocardial CMR tagging documented GLS and GCS deterioration 2 years after AVR (*p* < 0.03 for both), despite a recovery in LV ejection fraction and a shrinkage of LV dimensions [69]. In addition, Ungacta et al. demonstrated a reduction in posterior wall circumferential strain in patients with AR 6 months after AVR [70]. These data indicate that LV myocardial fibrosis in patients with AR is a flag of adverse remodeling that may conduce to further deterioration in the LV strain and weak prognosis after AVR.

## 5. Role of CMR in TAVI

CT is the gold standard imaging tool to assess the feasibility of TAVI. CT provides accurate annular sizing, determination of risk of annular injury and coronary occlusion, and co-planar fluoroscopic angle pre-procedural prediction. Further benefits of cardiac CT have also been demonstrated in the follow-up of TAVI for assessment of post-procedural complications, including identification of leaflet thickening [71,72].

However, CT requires contrast agent administration that may be high-risk in allergic patients and in subjects with chronic renal insufficiency, who make up a vast percentage of patients undergoing TAVI. The use of ionizing radiation in CT was not considered particularly relevant for the population involved in the intervention in the past; however, now, it represents a non-negligible issue with the extended indications to younger subjects with lower perioperative risk [53].

CMR may be a valid alternative to CT providing high-quality 3D multislice images without the administration of contrast agents and ionizing radiation. It offers the unique possibility of a one-stop-shop approach to not only assess the anatomical feasibility of TAVI, but to also provide a thorough analysis of the heart, including biventricular function, LV remodeling, myocardial fibrosis, and associated valvulopathies. Moreover, compared to CT, heart rate control is not a major concern because of the superior temporal resolution of CMR. Drawbacks of the use of CMR for TAVI include a longer study time, a greater patient collaboration, and underestimation of calcifications given that calcified tissue produces little signal.

**Figure 4:**
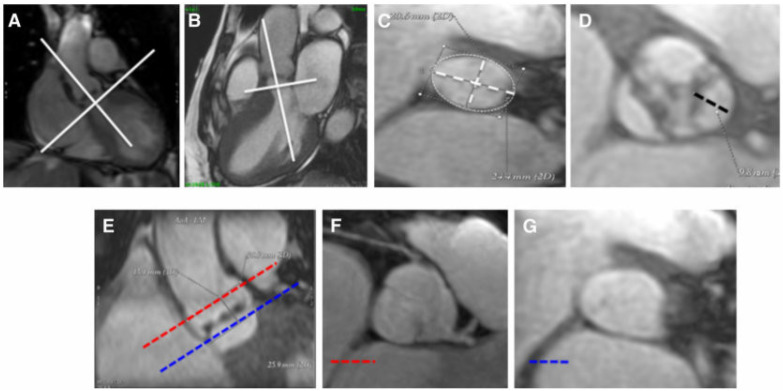
Approach for measurement of aortic annular size, aortic leaflet size, and coronary artery ostia using CMR. Assessment of aortic annulus (**A**–**C**): aortic annulus is defined as a virtual ring formed by joining the basal attachments of aortic valve leaflets. For aortic annulus, maximum diameter, minimum diameter, and area (white dot line) were traced in an orthogonal plane on the center line of the aorta achieved in oblique coronal and oblique sagittal views. Evaluation of leaflet length (**D**): the distance between the basal attachment and the apex of the leaflets (black dot line) is calculated. Measurement of coronary ostia height (**E**–**G**): a coronal view (**E**) and 2 short axes of the ascending aorta (**F**) and (**G**) at the level of the left main coronary ostium (red line) and aortic annulus (blue line) are obtained. The distance between these 2 lines is the coronary ostium height (adapted with permission from Elsevier [73], order number 5240390143830).

A complete evaluation with CMR of the aortic root, including assessment of annular size, aortic leaflet dimensions, and height of coronary artery ostia, is attainable and accurate when compared to cardiac CT [73,74] (Figure 4).

In order to perform these measurements, a CMR protocol pre-TAVI should include two long axis cine images of the aortic root and a stack of cine images acquired orthogonally to the above two planes, covering the entire aortic root.

Magnetic resonance imaging (MRI) can also be used to assess TAVI peripheral access route using CEMRA or, in patients allergic to contrast agents, a 3D-SSFP navigator-echo and ECG-gated (so-called whole heart) sequence for the thoracic aorta while a non-contrast-enhanced MR angiography can be used for aorto-iliac evaluation [36]. However, due to the limited assessment of calcification burden with MRI, risk stratifying for potential damage to access vessels is more effective with CT.

The identification of myocardial tissue characterization abnormalities with CMR allows prognostic stratification of patients before TAVI. Indeed, the presence of LGE in patients undergoing TAVI predicts higher cardiovascular disease related mortality [75].

Moreover, CMR is a powerful tool for the screening of association between amyloidosis and AS, which occurs in one of eight patients evaluated for TAVI [76]. Indeed, CMR with T1 mapping and LGE assessment is an ideal imaging technique in patients with a hypertrophic phenotype to raise the suspicion of cardiac involvement in amyloidosis [77].

The recent study by Nitsche et al. demonstrated that although patients with AS and cardiac amyloidosis were older and had worse clinical presentation (worse functional status, worse cardiac remodeling, higher circulating N-terminal pro-brain natriuretic peptide, and troponin levels), they had similar outcomes to those with lone AS [76].

However, there was a trend for higher mortality at 1 year in AS cardiac amyloidosis versus lone AS and other relevant clinical outcomes, including re-hospitalization for heart failure, functional class, and quality of life, which were not considered. Moreover, the study was limited to a 3-year follow-up, whereas cardiac amyloidosis may have an impact on longer-term outcomes [76].

Although the diagnosis of cardiac amyloidosis in symptomatic patients with severe AS should not preclude the consideration for TAVI, its identification with CMR is of importance as it may lead to consideration for pharmacological treatment [78].

In the post-intervention phase and follow-up, CMR can be valuable for the assessment of para-valvular aortic regurgitation, a condition associated with long-term mortality after TAVI [79]. TTE is the first-line technique to assess the prosthesis after TAVI. However, severity assessment of para-valvular regurgitation with TTE is difficult and dependent on patient factors (e.g., chest morphology, lung hyperinflation, suboptimal positioning, and valve calcific acoustic shadowing) [80]. On the contrary, CMR is a reproducible, accurate, and reliable method to assess para-valvular regurgitation severity after TAVI and is recommended in the presence of low quality or confidence in measured Doppler parameters and in cases of discordant quantitative and qualitative parameters and/or clinical data [80].

In the future, real-time CMR (RT-CMR) may be considered for guiding TAVI. Owing to an unlimited scan plane orientation and an unsurpassed soft-tissue contrast with simultaneous device visualization, RT-CMR could allow safe device navigation and offer optimal orientation for precise axial positioning. Non-contrast, radiation-free, and RT-CMR-guided TAVI has been successfully implanted in animals using dedicated conditional equipment [81], paving the way for future studies in humans.

## Data Availability

Not applicable.

## Abbreviations

2D	Two-dimensional
3D	Three-dimensional
4D	Four-dimensional
AR	Aortic regurgitation
AS	Aortic stenosis
AVA	Aortic valve area
AVD	Aortic valve disease
AVR	Aortic valve replacement
CEMRA	Contrast-enhanced MR angiography
CMR	Cardiac magnetic resonance
CMR-FT	CMR-derived myocardial feature tracking
CT	Computed tomography
ECG	Electrocardiogram
ECV	Extracellular volume
EDV	End-diastolic volume
GCS	Global circumferential strain
GLS	Global longitudinal strain
GRS	Global radial strain
GRE	Gradient echo imaging
HRF	Holodiastolic retrograde flow
LGE	Late gadolinium contrast-enhanced
LV	Left ventricle/ventricular
LVOT	Left ventricle outflow tract
MOLLI	Modified Look–Locker inversion recovery
MPR	Multiplanar reconstruction
MRI	Magnetic resonance imaging
PC	Phase contrast
RT-CMR	Real-time cardiac magnetic resonance
SSFP	Steady-state free precession
TTE	Transthoracic echocardiography
TEE	Transesophageal echocardiography
TAVI	Transcatheter aortic valve implantation
VENC	Velocity-encoding
